# Shared Major Metabolic Pathways and Potential Targeted Therapies in Malignancies and Systemic Lupus Erythematosus

**DOI:** 10.3390/biom16081206

**Published:** 2026-08-18

**Authors:** Jaron Dalgleish, Maurice Tohme, Michael D. Pisano, Wen-Hai Shao

**Affiliations:** 1 Doctor of Osteopathic Medicine Program, A.T. Still University, 800 W Jefferson St., Kirksville, MO 63501, USA; sa217175@atsu.edu (J.D.); sa216516@atsu.edu (M.T.); 2 Department of Microbiology and Immunology, Kirksville College of Osteopathic Medicine, A.T. Still University, 800 W Jefferson St., Kirksville, MO 63501, USA; michaelpisano@atsu.edu; 3 Department of Pharmacology, Microbiology and Immunology, College of Osteopathic Medicine, Xavier University, Cincinnati, OH 45207, USA

**Keywords:** cancer, SLE, metabolism, glycolysis, FAO, mitochondrial dysfunction, therapy

## Abstract

Systemic lupus erythematosus (SLE) is a multifaceted autoimmune disease characterized by immune tolerance breakdown, immune cell dysfunction, and chronic inflammation. Cancer is a serious health problem and the second leading cause of mortality worldwide. Emerging evidence underscores the key role of metabolic dysregulation and the association with immunity and immune-related complications in cancer and SLE. Enhanced glycolysis and OXPHOS have been repeatedly reported in both diseases. Metabolic reprogramming is common in cancer cells and immune cells of SLE patients. In many cases, cancer cells and B cells rely on fatty acid oxidation to generate energy. Accordingly, key enzymes in those processes are also upregulated. This review summarizes current findings on major common metabolic dysregulation in cancer and SLE, highlighting the interplay of metabolic disturbances, mitochondrial dysfunction and disease pathogenesis. Furthermore, we explore the potential of targeting metabolic pathways as a therapeutic strategy to mitigate organ damage and improve outcomes in patients with SLE or cancer. We will also discuss the hurdles and prospective developments in metabolism-targeted therapy. We hope this review inspires collaborative work between cancer researchers and SLE clinicians and facilitates clinical application of cancer-metabolism-targeted drug in SLE patients.

## 1. Introduction

Systemic lupus erythematosus (SLE) is a chronic autoimmune disorder caused by breakdown of immune tolerance, dysregulation of inflammatory responses, and immune cell hyperactivity [1,2]. SLE etiology is unknown, and pathogenesis is not fully understood. Therapeutic intervention is inadequate to control the disease; flares occur regardless of the advanced treatment [3]. Cancer is a leading cause of death globally, characterized by uncontrolled division of invasive abnormal cells that disrupt normal physiological functions [4]. Surgical removal of solid tumors, followed by chemo/radiotherapy is still the primary method [5]. Metabolic dysfunction plays a crucial role in the pathogenesis of cancer and SLE, though the specific mechanisms and their dysregulation differ [6,7,8] and the outcomes are often disparate. Glycolysis is found to be the primary metabolism in cancer. Increased glycolysis is observed in activated T and B cells in lupus patients [9]. On the other hand, T cell glycolysis is outcompeted by cancer cells in the tumor microenvironment (TME) [10]. Fatty acid synthesis is important for activating lupus T and B cell proliferation and autoantibody production and for cancer cell survival and proliferation [11,12]. Dysregulated fatty acid oxidation (FAO) contributes to immune cell differentiation and function in SLE. Cancer cells also rely on FAO for energy. Therefore, dysregulated common metabolic pathways exist in cancer patients and SLE cohort. Changes in cellular metabolism contribute to tumor transformation and progression, as well as SLE disease activity. Metabolic phenotypes can be exploited to predict prognosis in both diseases. Metabolic pathway targeting drugs that were initially developed for cancer patients may be redirected to treat patients with SLE. In this review, we will summarize major common metabolic pathways that contribute to the disease pathogenesis in patients with cancer or SLE. We will explore their specific functions in cells involving cancer survival, proliferation, and metastasis in line with functions in immune cell proliferation and activation in SLE. We will provide with prospect view of therapeutic targets that may benefit patients with cancer and patients with SLE. We hope to see more collaborative work between cancer researcher and SLE clinicians. Ultimately, cancer clinical drugs should benefit SLE patients and vice versa targeted therapy in SLE help understand immune cell regulation in cancer patients.

## 2. Glycolysis

**Figure 1:**
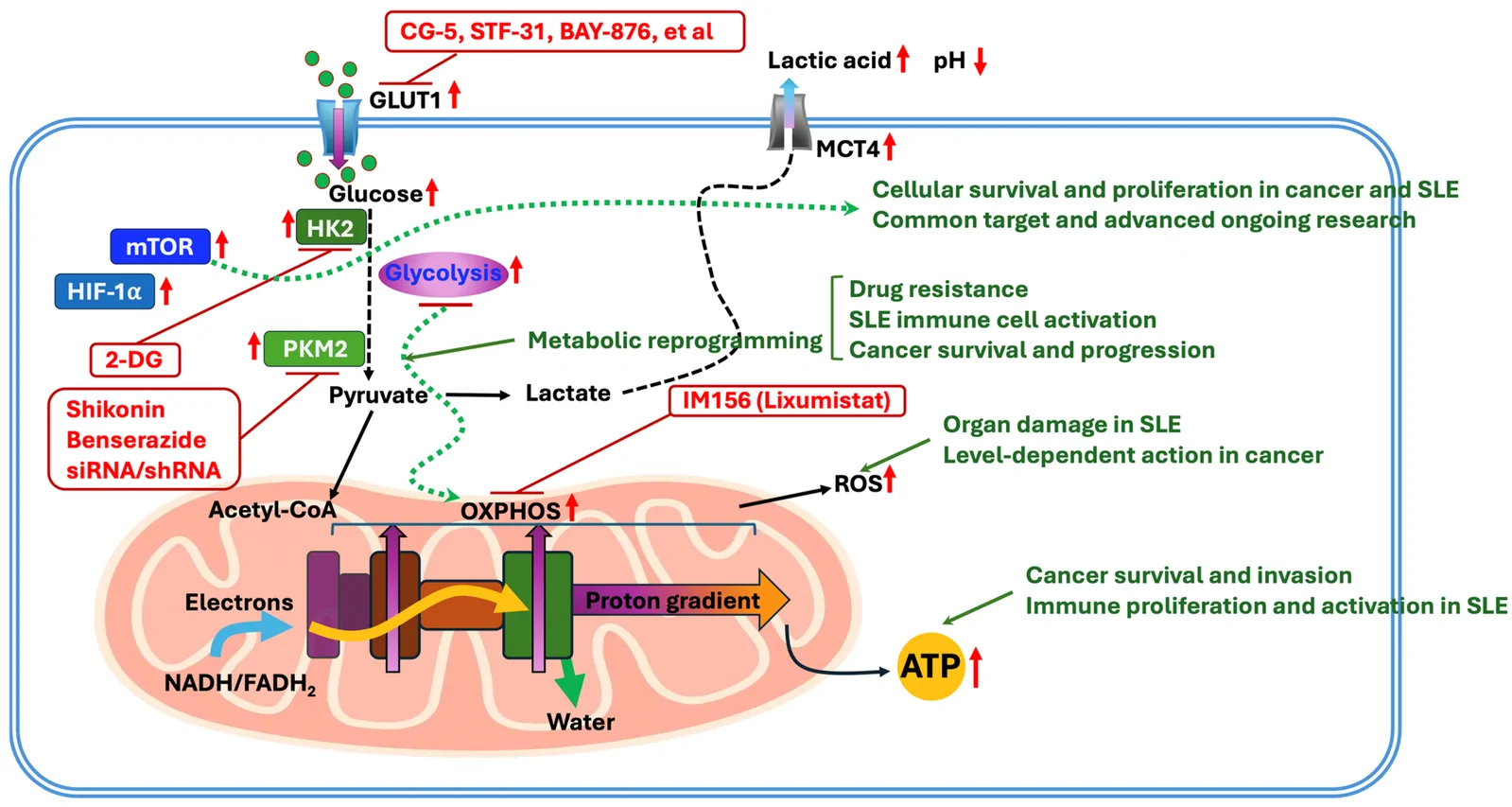
Glycolysis and targeted therapy in cancer and SLE. Cancer cells and immune cells in SLE patients rely on glycolysis for energy. They upregulate glucose transporter 1 and downstream enzymes (HK2 and PKM2). Cancer cells break glucose into lactate. Lactic acid is then excluded to the TME by monocarboxylate transporter (MCT4). Some cancer cells and immune cells in SLE patients rely on OXPHOS for energy for survival and during metabolic reprogramming. Increased glycolytic state is maintained via mTOR and HIF-1⍺. Inhibition of glycolysis leads to metabolic reprogramming to OXPHOS. Upward red arrows indicate upregulation. Downward red arrows indicate downregulation. Red boxes are inhibitors targeting specific metabolic processes. ATP, adenine triphosphatase; DCs, dendritic cells; GLUT1, glucose transporter 1; HIF-1⍺, hypoxia-inducible factor 1 alpha; HK2, type II hexokinase; mTOR, mammalian target of rapamycin; OXPHOS, oxidative phosphorylation; PKM2, M2 isoform of pyruvate kinase; ROS, reactive oxygen species; SLE, systemic lupus erythematosus.

Glycolysis is a universal metabolic pathway that takes place in the cytosol of almost all living cells. It involves a sequence of 10 enzymatic reactions that break down one molecule of glucose into two molecules of pyruvate [13]. The major function of glycolysis is to provide ATP for survival in the absence of oxygen or generate intermediates that fuel the biosynthesis pathway in the presence of oxygen [14]. Unlike normal cells, cancer cells primarily utilize aerobic glycolysis for energy, breaking down glucose into lactate even when oxygen is abundant. This inefficient glycolytic process is known as Warburg Effect [15]. It represents a prominent metabolic characteristic of malignant cells (Figure 1). Though the exact mechanism responsible for this metabolic alteration remains to be elucidated, several factors may contribute to the predominant glycolysis in cancer cells, including increased expression of glucose transporter 1 (GLUT1), enhanced glycolysis-processing enzymes, and suppressed mitochondrial respiration. In support of this notion, a recent meta-analysis of 17 studies with a total of 1795 patients found that GLUT1 overexpression has a significantly high prevalence in cancer tissues, particularly in urological, head and neck cancers [16]. The predominant M2 isoform of pyruvate kinase (PKM2) and type II hexokinase (HK2) are upregulated in tumors and they are crucial for tumor development and progresses [17,18,19]. Mitochondrial respiration defects in cancer cells have been broadly reported and can be attributed intrinsically to somatic mitochondrial DNA mutation and mitochondrial enzyme dysfunction and extrinsically altered oncogene-mediated mitochondrial respiratory suppression [20,21,22]. Nevertheless, cancer cell glycolysis rapidly generates energy and metabolic intermediates necessary for tumor growth, proliferation and invasion. To ensure their own glycolysis continues, tumor cell glycolysis generated lactic acid is actively excluded from the cell by monocarboxylate transporter (MCT) proteins. In line with this, lactate transporter MCT4 was reported positive in malignant tissues and significantly associated with reduced overall survival in malignant pleural mesothelioma (MPM) patients [23]. Accumulation of lactic acid in the TME leads to reduced extracellular pH and immunosuppression and facilitates a tumor-favorable TME [24,25]. In colorectal cancer cells, aerobic glycolysis-generated lactic acid stimulates the formation of vascular endothelial cells, which promotes tumor progression and metastasis [26]. The increased glycolytic state is in general maintained via mTOR (mammalian target of rapamycin) and HIF-1α (hypoxia-inducible factor 1 alpha) signaling [27,28]. Dysregulation of the mTOR pathway is frequently reported in many types of human tumors [29]. Overactivation of mTOR promotes cancer growth, metastasis, and invasion [30,31]. HIF-1α facilitates invasion, angiogenesis, metabolic reprogramming, and cell survival in a low-oxygen environment, leading to the growth and spread of tumors [32].

Myeloid-derived suppressor cells (MDSCs) are a group of heterogeneous cells derived from myeloid progenitor cells after pathological activation. MDSCs play crucial rules in immune-associated diseases, including autoimmune diseases and tumors [33]. Metabolic regulation in MDSCs is important for the enhancement of immunosuppressive activity, which is essential for tumor growth and immune escape, leading to poor clinical outcomes. Increased glycolytic activity was reported in MDSCs compared to normal myeloid cells [34]. This is further supported by increased GLUT1 expression during MDSC differentiation and markedly elevated intracellular glucose concentrations [35].

While glycolysis is considered the backbone of tumor cell metabolism, distinctly altered glucose metabolism is common in SLE (Figure 1). Dysregulation of glycolysis in lupus development exhibits a cell-dependent manner. T cells have a central role in the pathogenesis of SLE. Their dysregulation affects peripheral tolerance and induces inappropriate B cell activation [36]. Jin et al. found a significantly higher glycolytic capacity in SLE-CD4 T cells. Those CD4 T cells exhibit increased proliferation rates and elevated cytokine levels. Glycolysis of the SLE-CD4 T cells is also positively correlated with SLE disease activity and cytokines [37]. B cells are the central player in SLE; they secrete autoantibodies, cytokines, and present antigens. B cells become the major target in drug development [38,39]. Majority of activated B cells are glycolytic in SLE [40]. Consist with the findings from cancer cells, glycolytic genes HK2 and PKM2 are also elevated in SLE B cells. Upregulation of these genes correlates with increased production of inflammatory cytokines and autoantibodies [41,42]. Germinal center (GC) B cell differentiation is key to the production of pathogenic autoantibodies in SLE. GC B cell metabolic shift towards glycolysis is a central feature in SLE pathogenesis [43]. Dysregulation of mTOR and HIF-1α was also reported in SLE patients. mTOR, a key sensor of metabolic stress, is chronically activated in SLE patients in part due to increased reactive oxygen species (ROS) production and serves to increase glycolytic activity [44]. HIF-1α is stabilized by some inflammatory metabolites, such as succinate, which allows it to exert its effects even in normoxic conditions [45]. HIF-1α can then upregulate glycolytic enzymes and glucose transporters while simultaneously preventing pyruvate from being shuttled for oxidative phosphorylation by deactivating pyruvate dehydrogenase [45]. Pyruvate is now funneled into the lactate dehydrogenase pathway, allowing for regeneration of NAD+ for continued glycolysis at the expense of lactate accumulation [46].

In SLE patients, significantly increased MDSCs is positively correlated with the disease severity [47]. However, MDSCs can be both immunosuppressive and promotive in lupus pathogenesis, depending on the contexts examined. MDSCs from SLE patients exhibited significantly elevated arginase 1 (Arg-1) production and increased potential to promote Th17 differentiation in vitro in an Arg-1-dependent manner. Humanized SLE mouse model study revealed that MDSCs are essential for the induction of Th17 responses and the associated renal injuries, and the effect of MDSCs is Arg-1-dependent [48]. In contrast, studies with the Sanroque animal model of lupus showed that MDSCs induce the expansion of IL-10 secreting B10 B cells and decrease in the populations of effector B cells. Furthermore, infusion of MDSCs reduced anti-dsDNA levels and improved renal pathology in lupus mice, due to an expansion of regulatory B cells and a decrease in T follicular helper (T_FH_) cells, Th1 cells and Th17 cells. Interestingly, the expansion of regulatory B cells is completely blocked by N^G^ -monomethyl-l-arginine, a nitric oxide synthase (iNOS) inhibitor, indicating that MDSC-induced regulatory B cell expansion is iNOS-pathway-dependent, but not Arg1-pathway-dependent [49]. Arg1 and iNOS are key enzymes that metabolize L-arginine, but act as opposing biological markers [50]. This discrepancy is probably due to the heterogeneity of MDSCs. There are two main human subsets: CD15^+^CD66b^+^ granulocytic (G-MDSCs) and CD14^+^HLA-DR^−^ monocytic (M-MDSCs) cells. G-MDSCs consist of relatively immature and pathologically activated neutrophils, whereas M-MDSCs are inflammatory monocytes [51]. Mice have similar MDSC subsets but very different markers [52]. In both studies, subsets of MDSCs were not specified. On the other hand, improved nephritis in humanized lupus mice occurred due to MDSC depletion, but not infusion, which reflects an indirect effect of MDSCs. MDSC function in the Sanroque lupus mice is even more complex. Endogenous MDSCs drive autoimmunity by promoting plasma cell survival and CD4 T cell differentiation [53], but C57BL/6 mice MDSC infusion suppresses autoimmunity [49]. More studies are needed to further dissect the subset- and species-specific function of MDSCs.

## 3. Mitochondrial Dysfunction

Mitochondria plays a pivotal role in cellular metabolism and has been reported to be essential in immune response [54]. Oxidative phosphorylation (OXPHOS) is the final stage of cellular respiration, occurring in the inner mitochondrial membrane, where it produces the vast majority of ATP. The additional potential energy stored in the electrochemical gradient drives the transport of small molecules into and out of mitochondria [55]. Cancer cells exhibit heterogeneity in an energy source and can shift their reliance from glycolysis to mitochondrial OXPHOS. Recent studies have shown certain types of cancer, including leukemias, lymphomas, pancreatic ductal adenocarcinoma, upregulate OXPHOS instead of glycolysis to obtain their ATP to promote tumorigenic potential. This can occur even in the presence of active glycolysis [56]. Mitochondrial metabolism is crucial for tumor proliferation, survival, metastasis, and drug resistance. Enzymes and their mutations involve in tumor mitochondrial function have been reviewed elsewhere [57].

Mitochondria plays crucial roles in cancer stem cells (CSCs) in different aspects. CSCs are a special subset of tumor-initiating cells that are capable of self-renewing, bearing long-term of tumorigenicity, and generating more bulk tumor cells [58]. Mitochondrial biogenesis is required for the survival and propagation of CSCs. Inhibition of mitochondrial biogenesis markedly reduces OXPHOS and other independent signaling pathways that are normally required for the survival of CSCs [59]. Though the glycolytic favorable metabolic phenotype of CSCs has been reported in breast cancer cells [60] and in two carcinoma studies [61,62], other CSCs have demonstrated OXPHOS-dependent metabolism [63,64,65,66]. CSCs upregulate OXPHOS to meet the high energy demands, facilitating tumor growth, migration, and therapy resistance [67]. The discrepancy of CSC metabolic phenotypes highly depends on the origin of the cells, the isolation or depriving methods, and the TME, which actively shape metabolic niches through context-dependent interactions. CSCs in hypoxic, invasive or immune-modulating states favor glycolytic metabolism [68], while mesenchymal stem cells (MSCs) support CSC survival under stress by maintaining mitochondrial OXPHOS [69]. Regardless of the metabolic preference, mitochondrial biogenesis and structure is crucial in maintaining stemness and functionality of CSCs [67].

Mitochondrial dysfunction is also significantly associated with SLE disease activity, especially with renal involvement [70]. Mitochondrial ATP production and ROS are also closely associated with SLE pathogenesis [71]. Increased ROS production in immune cells, including T and B cells, dendritic cells (DCs), macrophages, and neutrophils, leads to disrupted mitophagy, and alterations in energy metabolism [72]. Higher mutation rate and lower repair efficacy of mitochondrial DNA (mtDNA) increase vulnerability to SLE. MtDNA in the cytosol or blood triggers severe inflammation and autoantibody production [72]. Significantly higher OXPHOS was also reported in SLE-CD4 T cells that demonstrated increased proliferation rates and elevated cytokine levels [37]. Japanese SLE cohort study revealed an OXPHOS signature in SLE memory B cells that is associated with the type I interferon signature, which is the dominant molecular signature found in SLE patients [73].

## 4. Fatty Acid Oxidation

**Figure 2:**
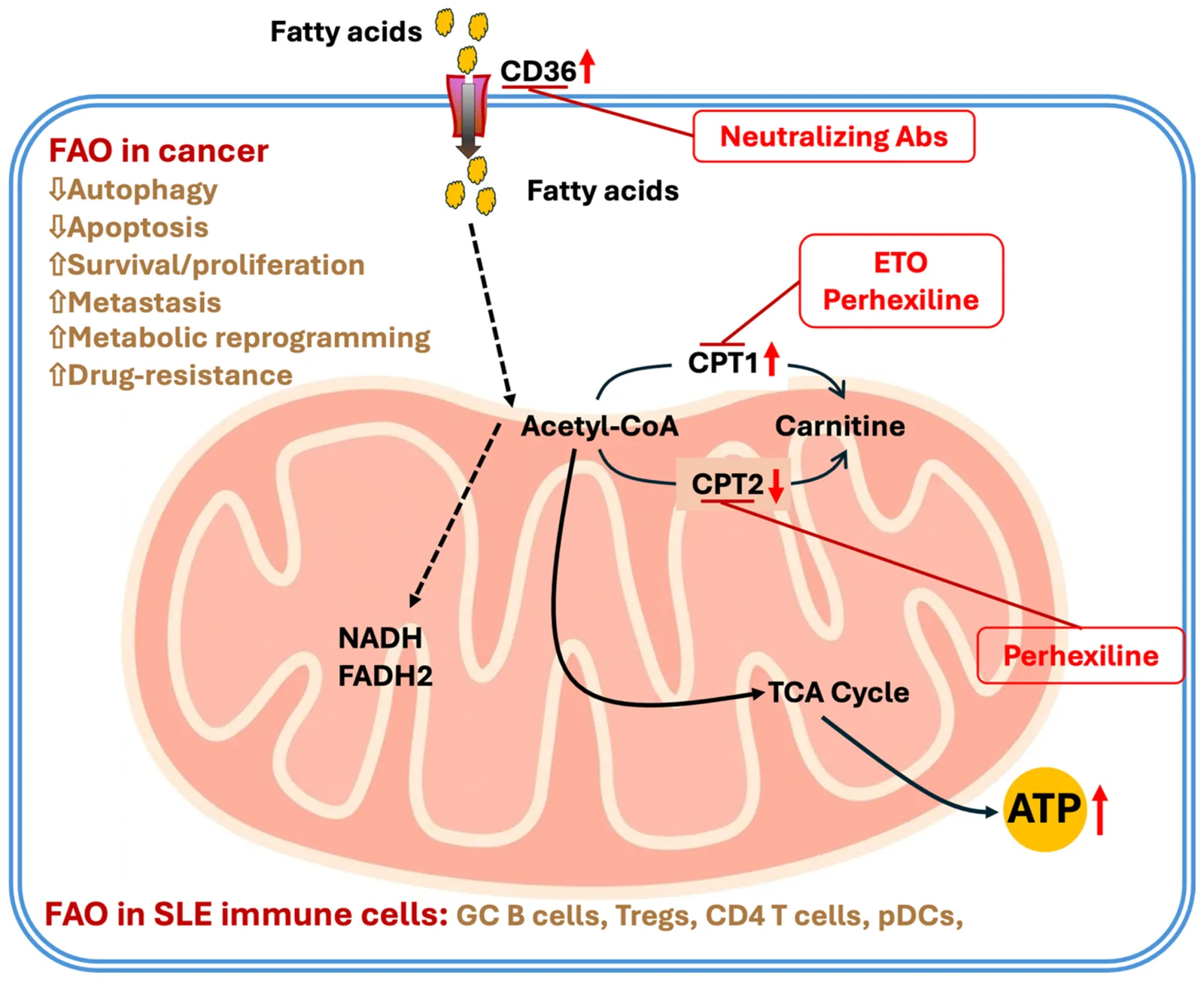
Fatty acid oxidation and its inhibition in cancer and lupus. Elevated levels of fatty acid receptor CD36 and CPT1, together with decreased CPT2 together contribute to enhanced FAO in cancer cells. B cells, especially GC B cells rely on FAO to generate energy. Upward red arrows indicate upregulation. Downward red arrows indicate downregulation. Red boxes are inhibitors targeting specific metabolic processes. CPT1, carnitine palmitoyltransferase 1; CPT2, carnitine palmitoyltransferase 2; ETO, etomoxir; FAO, fatty acid oxidation; GC, germinal center.

Fatty acid oxidation (FAO) is a process responsible for breaking down fatty acids into acetyl-CoA units in the mitochondrial aerobic process [74]. Under normal conditions, FAO is activated when the glucose energy supply is limited. However, FAO has been found to be enhanced and key FAO mediators are overexpressed in several types of malignant tumors (Figure 2). Diffuse large B cell lymphoma (DLBCL) utilizes FAO as the main energy source, and pharmacologic perturbation of this pathway is selectively toxic to this specific tumor subset [75]. Carnitine palmitoyltransferase 1C (CPT1C), located in the mitochondrial outer membrane, converts acyl-CoA to acyl-carnitine. It is frequently upregulated in lung tumors and its constitutive expression is associated with increased FAO [76,77]. Interestingly, CPT1C expression correlates inversely with mTOR pathway activation, contributing to rapamycin resistance in murine primary tumors [76]. Expression of CPT2, an enzyme that reforms acyl-carnitine back to acyl-CoA, is significantly lower in ovarian cancer (OC) cells than in non-malignant ovarian cells and normal tissues. This low expression of CPT2 is associated with a worse prognosis [78]. CD36, the fatty acid receptor, is expressed at high levels in human oral carcinomas and contributes to the initiation of metastasis [79]. Studies with OC cell lines and primary cells revealed a metabolic shift from glycolysis to FAO during cisplatin resistance and the FA uptake correlates linearly to the level of cisplatin resistance [80]. Overall, abnormal activation of FAO may inhibit cell autophagy, protect cancer cell from apoptosis, and promote cancer cell survival, metastasis, and drug resistance [77,81,82].

Pathogenic immune cells, especially B cells and antibody-secreting cells heavily rely on FAO for energy generation to drive autoimmune responses. Germinal center (GC) B cells are critical for generating isotype-switched high-affinity autoantibodies in SLE. Studies demonstrated that GC B cells primarily rely on FAO to generate energy [83], while Treg cells and memory CD4 T cells produce energy from OXPHOS and FAO [84] (Figure 2). Plasmacytoid DCs (pDCs) are the major source of IFN-I in SLE. The autocrine function of IFN-I in pDCs induces cellular metabolism changes characterized by enhanced FAO and OXPHOS. Direct inhibition of FAO prevents full pDC activation [85], indicating therapeutic potential of pDC targeted inhibition in SLE. Great advances of fatty acid metabolism in adaptive immunity have been made and summarized elsewhere [86]. However, immune cell FAO in SLE has not been exclusively explored, and current data is rather confusing—in some cases, even controversial.

## 5. Targeting Glycolysis in SLE and Cancer

Cancer cells and activated immune cells rely on glycolysis for energy needs. Glycolysis inhibition in cancer cells has long been recognized as a viable strategy for preventing cancer cell growth. Investigators have attempted to inhibit several steps in the glycolytic process. Pharmacological approaches have indeed demonstrated the effectiveness of this therapeutic strategy in cancer cells that include drugs targeting glycolytic enzymes and transporters (Figure 1). GLUT1 is a key regulator in tumor metabolic adaptation and has been demonstrated to be highly expressed in various cancer types, suggesting that GLUT1 is a fruitful target for cancer therapy. Current GLUT1 inhibition strategies include inhibitors or antibodies targeting specific regions of the protein (CG-5, BAY-876, et al.) [87] and disrupted *glut1* gene expression [88]. Majority of GLUT inhibitors is from selection of natural compounds. For example, Chan et al. screened ~64,000 small molecules and identified a second class compound of the pyridyl anilino thiazole, STF-31, that exhibits toxicity to renal cell carcinoma (RCC) with von Hippel-Lindau (VHL) tumor suppressor gene [89]. STF-31 specifically binds to GLUT1 to inhibit the glucose transport activity but doesn’t alter GLUT1 protein levels. STF-31-treated mice with RCC tumor xenografts displayed significantly decreased tumor growth compared to the tumor growth in untreated mice [89]. Another GLUT inhibitor, Compound G 5 (CG-5) specifically targets GLUT1 and GLUT5 [90]. Human colorectal cancer cells treated with CG-5 displayed dose-dependent energy restriction and increased apoptosis [91]. Although increased GLUT1 expression is observed in various types of cancers, GLUT1 is also a basal glucose transporter for all cell types. There are 14 members in the GLUT family with different substrate specificity and tissue expression [92]. We have not been able to target glucose entry in a tumor-specific manner for therapy due to the fact that cancer-specific GLUTs are not fully investigated. Nanoparticle technologies are nowadays a growing arena for drug delivery and tissue engineering for selective killing of cancerous cells [93], offering a promising solution for increasing drug selectivity, reducing significant side effects, allowing for multi-target inhibition, and enhancing therapeutic efficacy. Nanoparticle-directed GLUT1 inhibitors have been explored and summarized [87].

HK2 catalyzes the first enzymatic step of glycolysis. It is specifically overexpressed and exhibits particularly high enzymatic activity in the majority of malignant tumors [94]. 2-deoxy-D-glucose (2-DG), a glucose analog, is a competitive inhibitor of hexokinase [95]. 2-DG interacts with the tumor necrosis factor-related apoptosis-inducing ligand (TRAIL) and induces apoptosis in melanoma cell lines [96]. 2-DG also induces apoptosis in breast cancer cells when combined with hydroxychloroquine [97]. A phase I/II clinical trial on 20 cerebral gliomas patients with oral 2-DG followed by whole brain irradiation revealed excellent tolerance [98]. Beneficial therapeutic data are yet to be developed at this stage.

PKM2 is the final rate-limiting enzyme in the glycolytic pathway, generating pyruvate and ATP. PKM2 drives metabolic reprogramming in digestive system tumors [99]. Urothelial carcinomas predominantly over-express PKM2. Specific inhibition of PKM2 through siRNA or shRNA suppressed cell proliferation via increased apoptosis [100]. Shikonin is an active component extracted from Radix Arnebiae. Its antitumor effect has been demonstrated in nasopharyngeal carcinoma cells, hepatocellular carcinoma cells, and pancreatic ductal adenocarcinoma cells [101,102,103]. Zhang et al. showed that Shikonin suppresses PKM2-mediated aerobic glycolysis in esophageal squamous cell carcinoma [104]. Recently, a more specific PKM2 inhibitor was identified. Benserazide, an oral anti-Parkinson drug, exhibits high affinity to PKM2 and superior activity than Shikonin to inhibit PKM2. In vivo experiments showed markedly reduced tumor size with little body weight loss [105]. Other glycolytic enzyme inhibitors are summarized and exclusively discussed elsewhere [106].

Activated immune cells also rely on glycolysis for energy needs. Complete elimination of malignant cells depends on host immune cells, especially T cells. To solve this predicament, Inamdar et al. rescued DCs with microparticle-encapsuled fructose—1,6-bisphosphate (F16BP)—while blocking other cells with the rate-limiting 6-phosphofructo-2-kinase fructose-2,6-biophosphatase 3 (PFKB3) in glycolysis with PFK15 [107]. When F16BP is conjugated with melanoma peptide and poly(I:C), melanoma-inoculated mice exhibited with significant increased survival, which is associated with increased DC activation in the draining lymph nodes [107]. However, inhibiting glycolysis itself is insufficient to eradicate cancer cells due to the reason that cancer cells have the potential to adapt their metabolism to their environmental conditions. Shiratori et al. found that glycolytic suppression in multiple types of tumor cells leads to the reprogram of intracellular energy metabolism toward mitochondrial OXPHOS to ensure cellular survival [108].

Since cancer cells are highly dependent on glucose for energy and more susceptible to glucose deprivation compared with normal cells, it is reasonable to evaluate glucose deprivation as a cancer-preventative strategy. The inhibitory effects of low carbohydrate ketogenic diets (LCKDs) on tumor growth have been established in animal models and are also supported by a few clinical case reports [109]. Beneficial anti-inflammatory effects were reported in a female patient with juvenile idiopathic arthritis [110]. However, LCKD is not recommended for SLE patients. Animal studies revealed no beneficial effects (*Annals of the Rheumatic Diseases*, supplement). LCKD may also complicate other lupus-associated organ damages (*Summit Rheumatology*).

SLE disease management focuses on achieving a defined state of remission or low disease activity [111]. Recent advances in immunometabolism have also revealed glucose metabolic reprogramming in SLE [43]. Due to the complexity and interdependence of immunometabolic pathways, current therapeutic investigation targeting these pathways in lupus are mainly from the Morel group using animal models of lupus. They found that 2-DG reversed T follicular helper T_FH_ cell expansion and effector functions in lupus-prone mice. Most profoundly, adaptive transfer of 2-DG-reprogrammed T_FH_ cells protected lupus-prone mice from disease progression. Orthologs of genes responsive to 2-DG in murine lupus T_FH_ cells were found to be also overexpressed in T_FH_ cells of SLE patients [112]. The protective effect of glucose inhibition in lupus mice is also transferable through the gut microbiota. They further showed that fecal microbiota transfer (FMT) from 2-DG treatment protected lupus-prone mice of the same strain from development of nephritis and reduced autoantibody production [113]. Later on, they tried CG-5, an inhibitor of the GLUT, in the *sle1,2,3* triple-congenic lupus-prone mice and found reduced autoantibody production and GC B cell expansion, which is partially due to the CG-5 mediated inhibition of glycolysis in CD4 T cells [114].

## 6. Mitochondrial Function Manipulation

Mitochondria is fundamental in regulating cellular energy production, metabolism, calcium signaling, and apoptosis [115]. Targeting mitochondrial function has emerged as a promising therapeutic approach for SLE and cancer. Though, small molecule compounds and oligopeptides direct targeting mitochondrial fission and fusion proteins are available, but have not been investigated in animal models of cancer nor cancer patients [116]. IM156 (also known as Lixumistat), a biguanide mitochondrial protein complex 1 inhibitor of OXPHOS was the first-in-human study for dose escalation in patients with refractory advanced solid tumors [117]. It’s now being evaluated in combination with gemcitabine and nab-paclitaxel for the treatment of patients with advanced pancreatic cancer in a phase 1b trial (NCT05497778). In addition, blocking beta-oxidation through a small molecule inhibitor combined with cisplatin or carboplatin synergistically suppresses OC proliferation in vitro and growth of patient-derived xenografts in vivo [80]. IM156 treatment also significantly increased overall survival, reduced glomerulonephritis and inhibited B cell activation in the NZB/W F1 lupus-prone mice [118].

## 7. Combined Therapy with FAO Inhibition

FAO not only regulates cancer cell growth, proliferation, and metastasis, but also participates in cancer metabolic reprogramming and immunotherapy resistance. Therapeutic targets to inhibit FAO cause energy reduction and improve survival (Figure 2). Etomoxir (ETO) is an irreversible CPT1 inhibitor. Combined therapy with ETO in mouse orthotopic xenograft model showed prolonged survival with decreased glioblastoma stemness, and invasiveness [119]. Another study showed that ETO synergizes with Ezh2 in suppressing triple-negative breast cancer growth and lung metastasis in vivo [120]. ETO inhibited metabolic reprogramming also promotes cardiomyocyte proliferation and improves cardiac function post-myocardial infarction [121]. However, the clinical development of metabolic inhibition was halted due to significant hepatoxicity [122] and lack of enzyme selectivity [123]. Perhexiline, a CPT1A/CPT2 inhibitor, has been shown to alleviate platinum-based chemotherapy resistance of OC induced by NKX2-8 loss [124]. Little progress in cancer therapy has been made with these CPT1 inhibitors due to the lack of CPT isoform specificity and off-target toxicity [125]. CD36 has been pointed as a potential therapeutic target in cancer. The use of neutralizing antibodies to block CD36 causes almost complete inhibition of metastasis in immunodeficient or immunocompetent orthotopic mouse models of human oral cancer, with no side effects [79]. CD36-targeting antibody therapy may represent a promising direction in cancer treatment. Studies have shown additional benefits with CD36-targeting antibodies, including improved CD8 cytotoxic T cell function and reduced intratumoral Treg cells [126].

## 8. Discussion and Conclusions

Metabolic dysfunction has been demonstrated in cancer and SLE patients and animal models of the diseases for many years. Advanced research and discoveries have been made, especially in the field of cancer research. However, to date, few metabolism-targeted therapies have advanced to the bedside. Apparently, more research is still needed to understand the more depth implication of metabolic dysfunction in different stages of cancer development and subsets of cancer cells. In most cancers, suppressing cancer cell proliferation and survival while boosting immune responses spontaneously ensure a better outcome, except immune cell malignancy. However, immune cell activation and proliferation often require excessive energy consumption and increased metabolism. Metabolism inhibition in cancer patients also suppresses immune cell proliferation and activation, therefore dampening the overall outcome of metabolism-targeted therapy in cancers. Similar situation applies to metabolic intervention in SLE patients. Immune cells play differentiated roles in the pathogenesis at different stages of lupus disease development in different organs. Thus, an ideal metabolism-targeted drug should suppress pathogenic immune cells, such as autoantibody-secreting cells, activated T_FH_, Th17 cells, pDCs, and organ-infiltrating inflammatory cells, while sparing or enhancing functions of immune regulatory cells, such as Tregs, Bregs, and regulatory macrophages. Current drugs are not at the stage where they can distinguish between those cell subtypes. Another big hurdle in metabolism-targeted therapy is drug toxicity and off-targets. Metabolism is the primary function by which all cells extract and transform energy. Targeting metabolic steps will interfere with normal cell function. Metabolic dysfunction is not unique to cancer cells nor immune cells. As discussed earlier, isoforms of metabolic enzymes exist. Cancer cell and immune cell isoform-specific expression profile has not been exclusively studied.

An increasing number of studies has shown that cancer drug resistance is correlated with metabolic reprogramming. Cancer cells quickly adapt their metabolism to respond to treatments and environmental stress, a process known as metabolic transformation. However, cancer cell metabolic reprograming during therapeutic resistance development is still under-studied. Furthermore, cancer is not one disease—it is estimated that there are over 100 distinct diseases (according to the National Cancer Institute). Even a specific type of cancer is highly heterogeneous with unique metabolic adaptations. Similarly, SLE is not considered as a single disease but rather as a single syndrome with a set of signs, symptoms, or phenomena that occur together. Personalized metabolic profiling will not only enable the identification of metabolic alterations associated with disease initiation, progression, and treatment responses, but also guide optimal drug combination, duration, and dosage.

## 9. Therapy Development

More collaborative work and full-spectrum understanding of the differential function of metabolism in immune cells versus cancer cells are needed to better understand the complex yet distinct function. Cancer development and SLE disease progression are dynamic and responsive to medical intervention through metabolic reprogramming. Therefore, real-time metabolic imaging, as well as personalized glycolysis profile is essential for the successful disease management. Advanced metabolic research in SLE falls behind metabolic research in cancer. However, clinical results from trials in cancer patients should facilitate the quick translational application of metabolism-targeted drug to SLE patients. Current data indicate that metabolic monotherapy is likely less efficient. Combined therapy by targeting specific pathways in a cell type dependent manner may lead to better outcomes. Nanotechnologies has great potential in cell subtype specific therapy. Cell type differential combined treatments hold promise for future development.

## Data Availability

No new data were created or analyzed in this study. Data sharing is not applicable to this article.
